# Acute fibrinous and organizing pneumonia (AFOP): A case series

**DOI:** 10.5339/qmj.2024.qitc.11

**Published:** 2024-03-24

**Authors:** Shakeel Ahmed, Farhana Shora, Mousa Hussein, Mansoor Hameed, Merlin Thomas, Irfan Ul Haq, Mushtaq Ahmad, Tasleem Raza

**Affiliations:** 1 Department of Respiratory Medicine, Al Wakra Hospital, Al Wakra, Qatar; 2 College of Medicine, Qatar University, Doha, Qatar Email: sahmed30@hamad.qa; 3 Department of Clinical Medicine, Weill Cornell Medicine, Doha, Qatar; 4 Department of Chest, Hamad General Hospital, Doha, Qatar

**Keywords:** Organizing Pneumonia, Consolidation, Lung injury

## Introduction

Acute fibrinous and organizing pneumonia (AFOP) presents a unique response to acute lung injury characterized by interalveolar fibrin and organizing pneumonia. It typically affects the lower lobes of the lung and spans all age groups but is more prevalent in individuals aged 50–70 years.^1–3^

## Case #1

**Figure 1.**
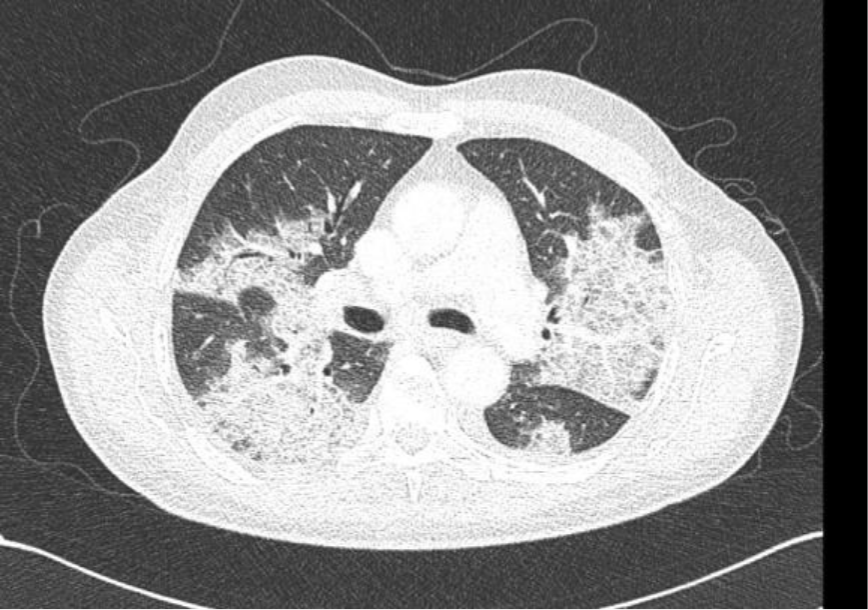
Case #1: CT image showing bilateral upper lobe consolidation.

A 71-year-old Indian male, former smoker with diabetes and coronary artery disease, presented with fever, cough, shortness of breath, and weight loss. He was hypoxic at 88% and showed left upper and mid-zone infiltrates on the chest radiograph and positive results for influenza A and H1N1. CT chest showed scattered consolidation and crazy paving. Confirmation of AFOP was made by transbronchial biopsies of the left upper lobe, and improvement was noted with oral steroids (Figure 1).

## Case #2

**Figure 2.**
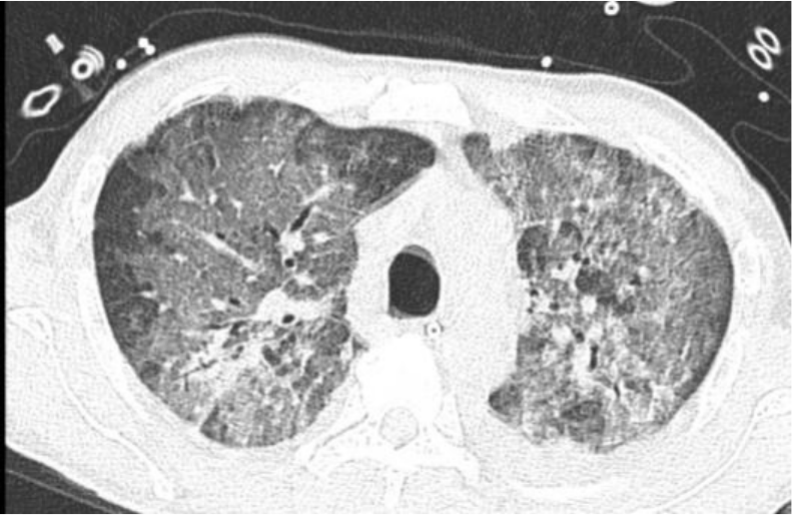
Case #2: CT image showing diffuse ground-glass changes.

A 52-year-old Filipino male with polyarthralgia, mouth ulcers, weight loss, vasculitis rash, and proximal muscle weakness with elevated creatine kinase and positive ANA in hypoxic respiratory failure showed no improvement with pulse steroids and intravenous immunoglobulins. CT chest revealed diffuse ground-glass changes and patchy consolidation, confirmed by open lung biopsy as AFOP, with a positive anti-melanoma differentiation-associated gene 5 indicative of dermatomyositis. Despite further immunosuppression, the patient did not survive (Figure 2).

## Case #3

**Figure 3.**
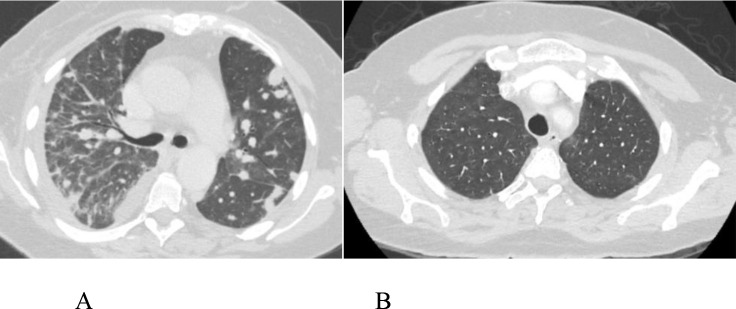
Case #3: CT image showing bilateral nodular opacities. (A) Before treatment. (B) After treatment.

A 51-year-old Canadian female with hypertension and a history of severe Covid-19 pneumonia presented with right-hand pain, erythema, swelling, fever, and cough. CT chest revealed well-defined cavitating nodular opacities. A CT-guided lung nodule biopsy confirmed AFOP. Oral steroids led to improvement, with a subsequent diagnosis of chronic myeloid leukemia after detecting a markedly elevated platelet count of 1,014 × 10^3^/Ul (Figure 3).

## Conclusion

AFOP is a clinicopathologically underdiagnosed disease with a variable appearance, suggesting that it may be more common than previously assumed. There is no established treatment consensus, resulting in a generally poor prognosis.

## Conflict of Interest

None for all Authors.
